# Analysis of the Association Between Motor and Anthropometric Variables with Change of Direction Speed and Reactive Agility Performance

**DOI:** 10.1515/hukin-2015-0069

**Published:** 2015-10-14

**Authors:** Tine Sattler, Damir Sekulić, Miodrag Spasić, Mia Perić, Ante Krolo, Ognjen Uljević, Miran Kondrič

**Affiliations:** 1 University of Ljubljana, Faculty of Sport, Slovenia.; 2 University of Split, Faculty of Kinesiology, Croatia.

**Keywords:** discriminant analysis, reactive strength index, conditioning capacities, morphology

## Abstract

There is an evident lack of studies examining the factors associated with reactive agility performances. The aim of this study was to evaluate the association between anthropometrics, body composition, jumping capacity, reactive strength, and balance with a stop-and-go change of direction speed (CODS) and reactive agility. The total sample comprised 39 male (body height: 182.95 ± 5.19 cm; body mass: 80.66 ± 7.69 kg) and 34 female (body height: 171.45 ± 6.81 cm; body mass: 61.95 ± 6.70 kg) college-level athletes (21.9 ± 1.9 years of age). The variables included body height, body mass, the percentage of body fat (BF%), balance as measured by an overall-stability index, the countermovement jump (CMJ), a reactive-strength index (RSI), stop-and-go reactive agility, and stop-and-go CODS. To define the associations between motor and anthropometric variables with CODS and reactive agility, the participants were clustered into three achievement groups based on their CODS and reactive agility performances. The ANOVA showed a significant difference between the CODS-based achievement groups for the CMJ (F test = 3.45 and 3.60 for males and females, respectively; p < 0.05), the RSI (F test = 6.94 and 5.29 for males and females, respectively; p < 0.05), and balance (F test = 3.47; p < 0.05 for males). In females, the reactive agility achievement groups differed significantly in the RSI (F test = 6.46; p < 0.05), the CMJ (F test = 4.35; p < 0.05) and BF% (F test = 4.07; p < 0.05), which is further confirmed by discriminant canonical analysis (Can R = 0.74; p < 0.05). The results confirm the need for independent evaluation and training for both CODS and reactive agility performance in sports.

## Introduction

Agility is defined as the ability to efficiently change the direction (and/or speed) of movement in response to stimuli. This is an important motor quality in sports where changes in direction are common (Delextrat et al., 2015; Lago-Penas et al., 2014; Vaczi et al., 2013). However, in real-life sport situations changes in direction are frequently made in response to unpredictable stimuli (Serpell et al., 2010; Sheppard et al., 2006). Consequently, the term “reactive agility” is used to describe a motor quality which consists of an effective change in direction in response to unpredictable (visual) stimuli, and to differentiate it from a pre-planned change of direction speed (change of direction speed – CODS) (Lockie et al., 2014; Sekulic et al., 2014a).

So far, the problem of reactive agility has mostly been investigated by researchers using a test involving sprinting on a “Y-shaped” course where the participants had to change their running direction only once during non-stop running (Lockie et al., 2013; Oliver and Meyers, 2009). However, from our point of view, this is a logical testing protocol in sports where agile manoeuvres consist of non-stop running. Meanwhile, other sports evidently demand more specialised reactive agility tests. This is particularly important in sports where a repeated multidirectional ‘stop-and-go’ reactive agility performance is common (i.e. tennis, basketball and handball). Recently, a novel method that is particularly useful for defining stop-and-go reactive agility was proposed and evaluated for its reliability and validity (Sekulic et al., 2014a).

Studies have frequently reported factors that influence different CODS performances (for an overview, see for example Spasic et al. (2013)). Yet, only a limited number of studies have investigated factors related to reactive agility (Henry et al., 2013; Scanlan et al., 2014; Spiteri et al., 2014). When investigating Australian rules footballers, authors found weak relationships between jumps and reactive agility performance (Henry et al., 2013). Among male basketball players, the morphological variables sprint and CODS had small to moderate correlations with reactive agility, while cognitive factors (response time, decision-making time) were moderately to strongly related to reactive agility (Scanlan et al., 2014). In a study of female basketball players, reactive agility did not correlate with strength variables (Spiteri et al., 2014). Interestingly, we found no study investigating balance in relation to reactive agility performance, although recent studies have demonstrated the crucial role of this variable in CODS (Sekulic et al., 2013). Moreover, no study has investigated factors associated with stop-and-go reactive agility.

The aim of this study was to evaluate the association between anthropometrics (body height, body mass), body composition (body fat percentage), vertical jumping capacity, reactive strength, and balance with the stop-and-go CODS and reactive agility performance in college-level athletes of both genders. Improving our understanding of these predictors will allow a more precise insight into the physical fitness attributes that directly determine reactive agility performance. It will also assure the more effective training of agility as well as sport selection for agility-saturated sports.

## Material and Methods

### Participants

The participants were college-age athletes (21.9 ± 1.9 years) involved in agility-saturated sports (i.e. football, basketball, volleyball and handball). The total sample comprised 39 males (body height: 182.95 ± 5.19 cm; body mass: 80.66 ± 7.69 kg) and 34 females (body height: 171.45 ± 6.81 cm; body mass: 61.95 ± 6.70 kg). All of them were well trained, in good health, and had no recent history of musculoskeletal disorders. All of the measurement procedures and potential risks were verbally explained to each participant and their informed consent was obtained. The Ethical Board of the Faculty of Kinesiology at the University of Split reviewed and approved the investigation.

### Variables and measurement

The sample of variables in this study comprised the measurement of body height, body mass, percentage of body fat (BF%), balance as measured by an overall-stability index (balance), a countermovement jump (CMJ), a reactive strength index (RSI), stop-and-go reactive agility, and stop-and-go CODS.

Body height and mass were assessed with a Seca stadiometer and a scale (Seca Instruments Ltd., Hamburg, Germany) using standard procedures, while body fat percentage (BF%) was measured using the MALTRON BF 900 analyser (Maltron International Ltd, Rayleigh, UK) (Peric et al., 2012). The CMJ and RSI were measured using the Optojump system (Microgate, Bolzano, Italy), a dual-beam optical device that measures ground contact and flight time during a jump or series of jumps. Balance was measured using a Biodex Balance System, BBS (Biodex Medical Systems, Shirley, NY, USA). Reactive agility and CODS were measured with original equipment recently presented and validated (Sekulic et al., 2014a).

The RSI is derived from the height jumped in a depth jump, and the time spent on the ground developing the forces required for that jump. The starting position for the depth jump involved the athlete standing upright on a 40-cm-high box. The participants were instructed to step off from the height and to jump up maximally, attempting to minimise the contact time (Ebben and Petushek, 2010).

The CMJ test began with an athlete standing in an upright position. A fast downward movement to about a 90° knee flexion was immediately followed by a quick upward vertical movement as high as possible, all in one sequence. The test was performed without an arm swing as the hands remained on the hips (Sattler et al., 2012).

The overall-stability (balance) index was an index of the average tilt in degrees from the centre of a platform. The higher the numerical value of the index, the greater the variability from horizontal positioning, that is, the greater instability while balancing on the platform. The participants were required to maintain an upright posture while keeping their arms by their sides and looking straight ahead at the Biodex LCD monitor approximately 0.3 m away. One practice trial was allowed before the three test trials. Each test trial lasted 20 s. The resistance level was set at number 9 on a scale ranging from 1 (least stable) to 12 (most stable) (Sekulic et al., 2013).

**Figure 1:**
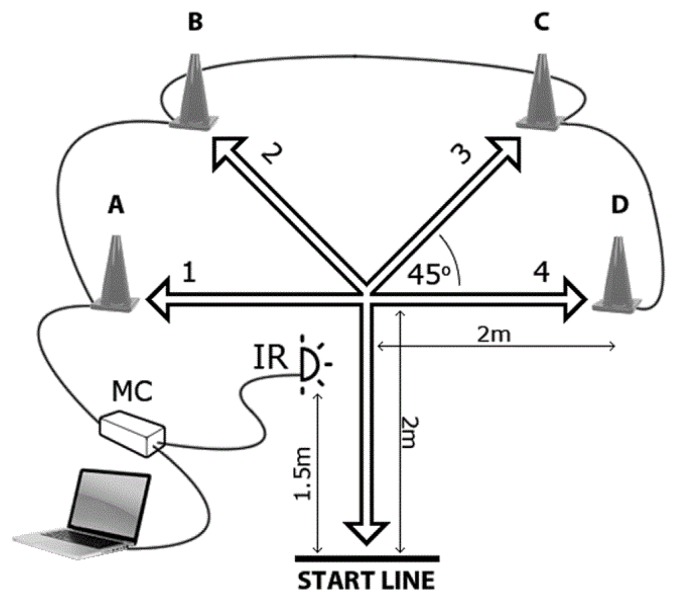
Testing of reactive agility and change of direction speed

The reactive agility test (reactive agility) was performed in the testing area shown in Figure 1. The participants began running from the start line when ready. Timing began the moment each athlete crossed the infrared (IR) signal. When an athlete broke the IR signal, a hardware module (microcontroller – MC) lit up one of the four LED lights placed inside 30-cm-high cones labelled A–D. An athlete had to assess which cone was lit, run to that particular cone, touch the top of it with their preferred hand, and return to the start line as quickly as possible. They then had to cross or step on the start line with their preferred leg, turn, and continue running over the next course. Each time an athlete broke the IR signal, the MC turned on one of the LED lights. A single-test trial consisted of three courses, and was completed when an athlete had broken the IR signal after returning from the third course. For the purpose of this study, all participants were tested using three equal scenarios (i.e. three testing trials), although they had no advance knowledge of them. The first scenario was B-D-B, the second was A-B-D, and the third was D-A-C. The best result was retained as the final score.

The CODS test was performed in the same testing area as reactive agility (Figure 1). Throughout this test, the testing scenarios were A-B-C, D-C-B, and C-B-A, and the participants knew them in advance. As for reactive agility, the timing began the moment each athlete broke the IR signal, and the best result was retained as the final score for each participant.

Throughout the practice trials, prior to the reactive agility and CODS the participants were made familiar with the testing procedures and established their most convenient manoeuvres. One-half of the participants completed the reactive agility test first, followed by the CODS test, while the other half performed the CODS test and then the reactive agility test. Standardised 3-minute pauses between the trials and tests were introduced for all participants.

All of the procedures were carried out indoors on a synthetic surface in a basketball gymnasium. The participants performed the tests wearing their choice of running shoes (excluding the balance testing, which was completed barefoot). Before testing, the participants completed a 15 min warm-up including jogging, lateral displacements, dynamic stretching and light jumping. To account for diurnal variation in fitness abilities, all of the tests were performed at the same time of the day (9 to 12 a.m.), and testing was done during December. Testing was conducted over two consecutive days. On the first day, the participants were tested on anthropometrics, the CMJ and the RSI. Balance, CODS and reactive agility were tested on the second day.

### Statistical Analyses

The reliability of the CODS and reactive agility measurements was checked via their intra-class coefficients (ICC).

To determine the association between CODS and reactive agility, Pearson’s correlations were calculated.

Since previous studies had not reported significant associations between predictors and reactive agility while using different types of correlational analyses, we decided to use a somewhat different statistical approach. For the purpose of this study, the athletes were divided into achievement groups according to their CODS and reactive agility performances. The low-achievement group compromised one-third of the athletes with the lowest performance (13 and 11 for males and females, respectively), the average-achievement group consisted of those athletes who were ranked between the 33^rd^ and 66^th^ percentile (13 and 11 for males and females, respectively), while the high-achievement group comprised one-third of the best performers (13 and 12 for males and females, respectively). Such grouping was performed independently for the CODS and reactive agility performances. Univariate differences between the achievement groups were determined using a one-way ANOVA with the Scheffe post-hoc follow-up test. To define multivariate differences between the groups, a discriminant canonical analysis was undertaken (Sattler et al., 2015). It allowed us to not only define the variables significantly associated with CODS and reactive agility performance, but also to define the hierarchy (i.e. relative importance) of the studied factors of influence. All analyses were stratified for genders.

Statistical significance was pre-determined at p < 0.05. Statistica, ver. 11 (Statsoft, Tulsa, OK) was used for all statistical calculations.

## Results

The reliability was appropriate for reactive agility (ICC of 0.81 and 0.84 for males and females, respectively) and strong for CODS (ICC of 0.91 and 0.94 for males and females, respectively). In general, females performed 14% better in CODS than in reactive agility (7.07 ± 0.58 and 6.11 ± 0.34 s for reactive agility and CODS, respectively), while males performed 13.5% better in the CODS than in reactive agility (6.50 ± 0.40 and 5.62 ± 0.41 s for reactive agility and CODS, respectively).

The correlations between CODS and reactive agility were significant (p<0.05) but moderate (r = 0.51 and 0.65; 25% and 42% of the common variance for males and females, respectively).

In males, significant univariate differences between the three achievement groups based on CODS performance were found for the CMJ, balance (i.e. the OSI index) and the RSI. Significant post-hoc differences were evidenced between low achievers and high achievers (for balance and CMJ), and between all three groups (for reactive strength) (Table 1).

When the females were grouped according to their CODS performance, the achievement groups significantly differed in the CMJ and the RSI. Significant post-hoc differences were found between low achievers and high achievers (for the CMJ), and between low achievers and the two other groups (for the RSI) (Table 1).

When the male athletes were divided into the three achievement groups according to their reactive agility performance, there was no single significant difference between the achievement groups. In the females, the reactive agility achievement groups differed significantly in the RSI, the CMJ and BF%, with significant post-hoc differences between low achievers and high achievers (for BF%) and low achievers and the two other groups (for the CMJ and the RSI) (Table 2).

The discriminant canonical analysis found significant multivariate differences between the CODS achievement groups in males (Can R = 0.91; p < 0.05). The RSI contributed most significantly to the differentiation of the groups. In total, 67% of the males were successfully classified (68% of low achievers; 60% of average achievers, and 77% of high- achievers). The discriminant analysis calculation did not reach statistical significance (Can R = 0.51; p > 0.05) when calculated between groups based on the reactive agility performance in males (Table 3).

In females, the RSI was the most significant discriminator between the groups based on CODS performance (Can R = 0.89; p < 0.05). In total, 60% of the females were successfully classified (i.e., 55%, 49% and 72% for the low-achievement, average-achievement and high-achievement groups, respectively). The CMJ, RSI and BF% most significantly contributed to discrimination of the female groups clustered according to their reactive agility performance (Can R = 0.74; p < 0.05), with 59% participants being successfully classified (60%, 48% and 67% of low achievers, average achievers and high achievers, respectively) (Table 3).

## Discussion

In comparison with previous reports, we can emphasise the high reliability of the CODS testing employed here (Lockie et al., 2014; Spasic et al., 2013; Sporis et al., 2010). The reliability parameters we found for reactive agility are similar to those previously reported for a Y-shaped-based reactive agility test and a recently presented general stop-and-go test (Sekulic et al., 2014a; Serpell et al., 2010). As a result, we can highlight the proper consistency of the tests we employed.

Differences between CODS and reactive agility are similar in both genders (i.e. about a 15% better performance in CODS), which is in accordance with previous findings (Sekulic et al., 2014a). Therefore, it seems that regardless of the gender, duration of the test, and movement template, the 15% difference should be considered as the average difference between reactive and non-reactive stop-and-go agility performances.

Previous studies that investigated a Y-shape reactive agility test and corresponding CODS noted practically a “null-correlation” between these two qualities (Serpell et al., 2010), while we found statistically significant correlation between CODS and reactive-agility. It is mostly explainable by the fact that our respected colleagues investigated CODS and reactive-agility performance throughout the tests consisted of non-stop running, whereas we observed stop-and-go CODS and reactive agility performances. However, the unexplained part of the variance in our study (i.e. 66% and 75% for males and females, respectively) is almost certainly related to other qualities such as cognitive capacities, and/or perceptual and reactive capacities, as already suggested (Scanlan et al., 2014; Serpell et al., 2010).

The findings of an evident influence of jumping capacity (i.e. CMJ) and reactive strength (i.e. RSI) on CODS performance directly support the results of previous studies where investigators identified those qualities as being significantly related to different CODS (i.e. non-reactive) performances (Spasic et al., 2013; Sekulic et al., 2013). This is mostly explained by the similar physiological background of jumping, reactive strength and CODS (i.e. all three performances require the intensive involvement of the fast-twitch muscle fibres) (Sekulic et al., 2013).

It seems that the importance of balance in CODS is only characteristic for males. Namely, to the best of our knowledge, none of the studies conducted so far has reported a notable association between balance and CODS in females (Sekulic et al., 2014b; Sekulic et al., 2013; Spasic et al., 2013). This is explained by two key issues. First, females are advanced in balance capacity over males and, second, females generally lack other motor qualities known to be important determinants of COD (i.e. power, speed, etc.) (Sekulic et al., 2013).

In females, the negative influence of BF% is evidenced for the reactive agility performance. This is almost certainly related to the fact that female athletes notably differ in BF%, and body fat is ballast-mass which directly alters a reactive agility performance. However, such an association between BF% and performance is not evidenced for CODS. Most likely, since we studied experienced female athletes, all of them were able to adapt their locomotion form and running technique according to the necessary change of direction (i.e. shorten their step, lower their centre of mass etc.). Therefore, the possible negative influence of body fat on the CODS testing is almost certainly diminished by proper adaptation of the running technique. Yet, this is only possible during CODS because of their prior knowledge of the movement scenario.

The differences between genders with regard to the associations which exist between CODS and reactive agility (i.e. 25% and 42% of the common variance for males and females, respectively) are almost certainly related to the differential influence of the studied motor qualities on CODS and reactive agility performance. Briefly, in the females we observed certain similarities in the structure of the discriminant roots calculated for reactive agility and CODS, while the discriminant analysis performed for reactive agility did not achieve statistical significance in the males.

In recent studies, reactive agility has been found to be weakly correlated with strength variables (Henry et al., 2013; Scanlan et al., 2014; Spiteri et al., 2014). Therefore, our results of a significant association of strength and power indices (i.e. the CMJ and the RSI) with reactive agility in females do not agree with previous findings. However, the difference between the reactive agility performances once again explains certain disagreement in the findings. In brief, previous studies used tests that consisted of only one change of direction (i.e. Y-shape tests) (Henry et al., 2013; Scanlan et al., 2014; Spiteri et al., 2014). Meanwhile, our reactive-agility test consisted of several changes of direction where power and strength qualities (CMJ or RSI) were repeatedly challenged (i.e. each stop-and-go moment throughout the test was practically an eccentric-changing-to-concentric-contraction template).

## Conclusion

This study indicated the importance of jumping capacity and reactive strength for stop-and-go CODS performance in male and female athletes. Therefore, training programmes aimed at improving stop-and-go CODS should include training modalities known to be efficient in the development of this conditioning capacity (plyometrics).

We may suggest the introduction of different balance exercises in order to potentially improve CODS in males. This would be a particularly convenient training method for improving CODS in those athletes who have advanced power capacities.

There are indices that an improvement in reactive agility among females should be expected as an effect of a simultaneous decrease of the body fat percentage and an improvement in the reactive strength and vertical jumping performance. However, coaches should be aware of the varying effects of some types of training modalities. For example, extensive aerobic endurance training, although highly effective in reducing BF%, may have a possible negative impact on strength abilities (i.e. jumping capacities and reactive strength).

The variables observed in this study were not found to be related to stop-and-go reactive agility performance in males. Therefore, future studies should explore additional factors potentially associated with such performances in male athletes, such as cognitive qualities, perceptual and reactive capacities, decision-making time, etc.

## Figures and Tables

**Table 1:** Univariate differences between the achievement groups based on change of direction speed performance

		High achievers	Average achievers	Low achievers	F-test
	
BH (cm)	Males	185.17±5.37	182.97±3.95	181.18±4.60	2.54
	Females	169.88±6.98	175.38±5.45	169.13±6.88	3.25
BM (kg)	Males	82.17±8.42	79.03±4.40	79.28±7.01	1.35
	Females	59.57±7.21	65.13±6.24	60.88±6.27	2.63
BF% (%)	Males	12.94±3.69	11.48±2.21	14.00±3.48	2.80
	Females	23.90±2.11	23.68±4.66	22.00±2.30	1.14
CMJ (cm)	Males	36.47±4.45 §	33.21±4.44	31.11±2.72 ¥	3.45*
	Females	27.78±4.11 §	25.06±5.04	22.93±3.18 ¥	3.60*
RSI (index)	Males	39.19±3.67 §,¶	33.43±4.02 ¥,§	35.79±3.89 ¥, ¶	6.94*
	Females	25.71±2.80 §	25.13±4.32 §	21.61±3.67 ¶,¥	5.29*
OSI (index)	Males	1.01±0.39 §	1.44±0.61	1.58±0.54 ¥	3.47*
	Females	1.05±0.55	0.98±0.25	1.01±0.21	1.32

Data are presented as Means ±SD; F-test – Analysis of the variance F test value; BH – body height; BM – body mass; BF% – percentage of body fat; CMJ – countermovement jump; RSI – reactive strength index; OSI – overall stability (balance) index;

¥ significantly different from high achievers;

¶ significantly different from average achievers;

§ significantly different from low achievers:

* denotes significant differences

**Table 2:** Univariate differences between the achievement groups based on reactive agility performance

		High achievers	Average achievers	Low achievers	F-test
		
BH (cm)	Males	183.97±4.34	182.00±3.50	184.08±5.18	0.44
	Females	172.88±7.81	172.50±6.44	169.00±6.28	1.48
BM (kg)	Males	81.76±6.24	79.03±5.20	81.45±9.08	0.91
	Females	65.29±7.36	60.75±6.86	60.25±5.63	2.24
BF% (%)	Males	12.03±2.05	12.65±3.55	13.70±3.89	0.55
	Females	21.08±2.54 §	23.87±3.35	24.50±3.57 ¥	4.07*
CMJ (cm)	Males	33.41±3.14	36.8±3.93	33.83±3.39	2.79
	Females	26.21±4.30 §	26.78±3.40 §	22.12±2.01 ¥,¶	4.35*
RSI (index)	Males	35.79±4.76	35.21±5.23	35.12±3.12	1.55
	Females	25.67±4.04 §	24.28±5.50 §	21.78±1.46 ¥,¶	6.46*
OSI (index)	Males	1.43±1.11	1.54±0.99	1.25±0.67	3.02
	Females	0.89±0.31	0.97±0.21	0.88±0.19	1.68

Data are presented as Means ± SD; F-test – Analysis of the variance F test value; BH – body height; BM – body mass; BF% – percentage of body fat; CMJ – countermovement jump; RSI – reactive strength index; OSI – overall stability (balance) index;

¥ significantly different from high achievers;

¶ significantly different from average achievers;

§ significantly different from low achievers:

* denotes significant differences

**Table 3:** Multivariate differences between the achievement groups based on change of direction speed performance and reactive agility performance

	Females				Males			
	
	Change of direction speed		Reactive agility		Change of direction speed		Reactive agility	
	
	Root 1	Root 2	Root 1	Root 2	Root 1	Root 2	Root 1	Root 2
	
BH (cm)	−0.07	−0.31	0.19	−0.75	0.06	−0.31	−0.45	0.75
BM (kg)	0.04	−0.83	0.17	−0.41	−0.04	−0.83	−0.45	0.41
BF% (%)	−0.01	−0.65	−0.47	−0.48	0.01	−0.65	0.07	0.47
CMJ (cm)	0.28	0.10	0.61	0.32	−0.15	0.09	−0.28	−0.31
RSI (index)	0.68	−0.03	0.51	0.28	−0.72	−0.03	−0.47	−0.27
OSI (index)	0.08	−0.29	0.02	−0.29	−0.07	−0.32	0.55	0.41
C: High achievers	2.08	0.33	0.67	0.50	−2.46	0.32	−0.31	−0.50
C: Average achievers	0.48	−0.52	0.31	−0.35	−0.55	−0.52	−0.64	0.35
C: Low achievers	−2.25	0.17	−1.08	−0.10	2.64	0.17	1.04	0.10
Can R	0.89	0.36	0.74	0.35	0.91	0.36	0.51	0.39
WL	0.18	0.87	0.34	0.88	0.13	0.86	0.49	0.41
p	0.01	0.80	0.04	0.81	0.00	0.80	0.61	0.80

BH – body height; BM – body mass; BF% – percentage of body fat; CMJ – countermovement jump; RSI – reactive strength index; OSI - overall stability (balance) index; Root – structure of the discriminant root; C – position of the centroid; Can R – canonical coefficient of correlation; WL – Wilks’ Lambda; p – level of significance

## References

[b1-jhk-47-137] Delextrat A, Grosgeorge B, Bieuzen F (2015). Determinants of performance in a new test of planned agility for young elite basketball players. Int J Sports Physiol Perform.

[b2-jhk-47-137] Ebben WP, Petushek EJ (2010). Using the reactive strength index modified to evaluate plyometric performance. J Strength Cond Res.

[b3-jhk-47-137] Henry GJ, Dawson B, Lay BS, Young WB (2013). Relationships between reactive agility movement time and unilateral vertical, horizontal and lateral jumps. J Strength Cond Res.

[b4-jhk-47-137] Lago-Penas C, Rey E, Casais L, Gomez-Lopez M (2014). Relationship between performance characteristics and the selection process in youth soccer players. J Hum Kinet.

[b5-jhk-47-137] Lockie RG, Jeffriess MD, McGann TS, Callaghan SJ, Schultz AB (2014). Planned and reactive agility performance in semi-professional and amateur basketball players. Int J Sports Physiol Perform.

[b6-jhk-47-137] Oliver JL, Meyers RW (2009). Reliability and generality of measures of acceleration, planned agility, and reactive agility. Int J Sports Physiol Perform.

[b7-jhk-47-137] Peric M, Zenic N, Mandic GF, Sekulic D, Sajber D (2012). The reliability, validity and applicability of two sport-specific power tests in synchronized swimming. J Hum Kinet.

[b8-jhk-47-137] Sattler T, Sekulic D, Esco MR, Mahmutovic I, Hadzic V (2015). Analysis of the association between isokinetic knee strength with offensive and defensive jumping capacity in high-level female volleyball athletes. J Sci Med Sport.

[b9-jhk-47-137] Sattler T, Sekulic D, Hadzic V, Uljevic O, Dervisevic E (2012). Vertical jumping tests in volleyball: reliability, validity, and playing-position specifics. J Strength Cond Res.

[b10-jhk-47-137] Scanlan A, Humphries B, Tucker PS, Dalbo V (2014). The influence of physical and cognitive factors on reactive agility performance in men basketball players. J Sport Sci.

[b11-jhk-47-137] Sekulic D, Krolo A, Spasic M, Uljevic O, Peric M (2014a). The development of a new stop‘n’go reactive agility test. J Strength Cond Res.

[b12-jhk-47-137] Sekulic D, Spasic M, Esco MR (2014b). Predicting agility performance with other performance variables in pubescent boys: a multiple-regression approach. Percept Motor Skill.

[b13-jhk-47-137] Sekulic D, Spasic M, Mirkov D, Cavar M, Sattler T (2013). Gender-specific influences of balance, speed, and power on agility performance. J Strength Cond Res.

[b14-jhk-47-137] Serpell BG, Ford M, Young WB (2010). The development of a new test of agility for rugby league. J Strength Cond Res.

[b15-jhk-47-137] Sheppard JM, Young WB, Doyle TL, Sheppard TA, Newton RU (2006). An evaluation of a new test of reactive agility and its relationship to sprint speed and change of direction speed. J Sci Med Sport.

[b16-jhk-47-137] Spasic M, Uljevic O, Coh M, Dzelalija M, Sekulic D (2013). Predictors of agility performance among early pubescent girls. Int J Perforf Anal Sport.

[b17-jhk-47-137] Spiteri T, Nimphius S, Hart NH, Specos C, Sheppard JM, Newton RU (2014). The contribution of strength characteristics to change of direction and agility performance in female basketball athletes. J Strength Cond Res.

[b18-jhk-47-137] Sporis G, Jukic I, Milanovic L, Vucetic V (2010). Reliability and factorial validity of agility tests for soccer players. J Strength Cond Res.

[b19-jhk-47-137] Vaczi M, Tollar J, Meszler B, Juhasz I, Karsai I (2013). Short-term high intensity plyometric training program improves strength, power and agility in male soccer players. J Hum Kinet.

